# A high figure of merit of phonon-polariton waveguide modes with hbn/SiO_2_/graphene /hBN ribs waveguide in mid-infrared range

**DOI:** 10.1016/j.heliyon.2024.e26727

**Published:** 2024-02-23

**Authors:** Zhou Sheng, Liu Yue, Yue Zhao, Gao Jin, Qiang Zhang, Shufang Fu, Xiangguang Wang, Xuan Wang, Xuanzhang Wang

**Affiliations:** a Department of Basic Courses, Guangzhou Maritime University, Guangzhou, 510725, China; b College of Science, Jiamusi University, Jiamusi, 154000, China; c *Key Laboratory for Photonic and Electronic Bandgap Materials*, *Chinese Ministry of Education, and School of Physics and Electronic Engineering*, *Harbin Normal University*, *Harbin*, 150025, *China*; d Key Laboratory of Engineering Dielectrics and Its Application, Ministry of Education, Harbin University of Science and Technology, Harbin, 150080, China

**Keywords:** Mid infrared range waveguide, Figure of merit, Hexagonal boron nitride, Hyperbolic phonon polariton

## Abstract

Natural hyperbolic materials can confine electromagnetic waves at the nanoscale. In this study, we propose a waveguide design that combines a high quality factor (FOM) with low loss, utilizing hexagonal boron nitride and graphene and gold substrate. The waveguide consists of a dielectric rib with a graphene layer sandwiched between two hBN ribs. Numerical simulations demonstrate the existence of two guided modes in the proposed waveguide within the second reststrahlen band (1360.0 cm^−1^<ω < 1609.8 cm^−1^) of hBN. These modes are formed by coupling the hyperbolic phonon polariton (HPhP) of two hBN rib in the middle dielectric rib and are subsequently modulated by a graphene layer. Interestingly, we observe variations in four transmission parameters, namely effective length, figure of merit, device length, and propagation loss of the guided modes, with respect to the operation frequency and gate voltage. By optimizing the waveguide's geometry parameters and dielectric permittivity, the modal properties were analyzed. Simulation results indicate that optimizing the waveguide size parameters enables us to achieve a high *FOM* of 4.0 × 10^7^. The proposed waveguide design offers a promising approach for designing tunable mid infrared range waveguides on photonic chips, and this concept can be extended to other 2D materials and hyperbolic materials.

## 1 Introduction

The swift development of optical technology has resulted in an increasing demand for small optical devices that offer high propagation and quality [1]. The manipulation of guided electromagnetic waves is of paramount importance in various fields, including nanophotonics, communications, analog computing, and integrated optics [2,3]. Optical waveguides can be categorized into two types: passive and active. Passive waveguides, such as parallel plate waveguides, metallic rectangular waveguides, metallic circular waveguides, slot lines, striplines, photonic crystal waveguides, and ribbon waveguides, have been widely utilized for interconnecting with external power sources, testing, and packaging purposes [4]. Waveguides are a popular choice for nanophotonic systems due to their ability to confine light within the metallic walls of the waveguide through total internal reflection (TIR) [5], the photonic bandgap (PBG) effect [6,7] and guided wave by metallic media (GMM) principle [8].

Active optical waveguides are a type of waveguide that can manipulate and control light signals. Xu conducted guided and surface waves in slab waveguides [9]. Tu depicted a mid-IR waveguide comprising a multilayer Gr/hBN structure and a Si nanowire. An unusual finding was observed, with propagation length (*L*_*p*_) and the normalized mode area (*A*_*m*_) decreasing with increaseing the Fermi energy (*E*_*F*_) [10]. Su presented a Si_3_N_4_ waveguide with a significant modulation bandwidth of around 80 GHz [11].

Conventional waveguides face challenges in achieving broad operation bandwidth, compact size, low power consumption, and efficient modulation through external stimulus [12]. Additionally, the diffraction limit imposes restrictions on the size of optical elements within these waveguides, limiting them to the wavelength scale and severely hindering device miniaturization. In the case of direct graphene modulators, another specific challenge arises from the limited absorption of a monolayer. However, this challenge can be addressed by integrating graphene with an optical waveguide. This integration significantly enhances the interaction length by promoting coupling between graphene and evanescent waves.

Fortunately, electromagnetic surface waves (SWs) are highly located at the material interface with deep subwavelength and local-field enhancement characteristics, such as surface plasmon polaritons (SPPs) [13], hyperbolic phonon polaritons (HPhPs) [14], etc. HPhPs are supported within hyperbolicity media (HM) where the permittivity along orthogonal axes is of opposite sign, such as hexagonal boron nitride (hBN) [15,16]. The HM dispersion relation exhibits a hyperbolic form in the reststrahlen frequency range, allowing for the support the very short wavelength and the ability to surpass the diffraction limit [17]. HM find wide applications, including thermal energy [18], biosensing [19], extraordinary radiative heat transfer [20], and the exploration of exotic photonic properties [21].

Graphene (Gr) has garnered considerable interest for its remarkable electrical and optical properties. The optical absorption properties of graphene can be controlled through electrical gating. By adjusting the applied gate voltage to modify the electronic Fermi level, it becomes possible to deliberately alter the optical transitions of graphene. As a result, graphene holds potential as a viable active medium for an optical electroabsorption modulator [22]. Graphene has been extensively studied in various optoelectronic fields, such as photodetectors [23], polarizers [24], optical modulators [25], and biosensors [26]. Moreover, the optical properties of graphene can be effectively manipulated by the external fields [27]. Graphene has the characteristics of dynamic tunability and low optical loss. Graphene-on-waveguide photonics have shown great potential for photonic integrated circuits applications [28]. Gaphene waveguides have been demonstrated to effectively transfer SPPs with low loss, long lifetime, and high confinement [29]. Moreover, graphene has been shown to provide superior light confinement to the device compared to other materials. Gr plasmons have attracted significant interest for their capability to confine light modes at scales significantly smaller than the wavelength in the air [[30], [31], [32]]. Using graphene as a component of the optical waveguide can offer a significant modulation depth. Dielectric loaded graphene plasmon waveguide (DLGPW) has been introduced and studied [33,34]. By adjusting the Fermi energy level, the propagation characteristics of fundamental mode could be flexibly tuned [[35], [36], [37]].

In 2021, Ma et al. devised a hybrid plasmonic rib waveguide for refractive index sensing [38]. The paper discusses the propagation loss spectra of four different types of high voltage pulses in microwave, each with distinct parameters. Furthermore, the study focuses on investigating the homogeneity and surface sensing performance of grid-based sensor structures. In Guang xin using low temperature nano infrared imaging to study propagation in isotopically pure hBN and naturally abundant α-MoO_3_ crystals, beyond the highly restricted conventional polaron model [7]. In 2022, Tu et al. conducted an analysis of hybrid plasmon-phonon-polariton modes in hBN/Gr/hBN stacks for mid-infrared waveguiding. The waveguide structure's guiding mode is formed through the coupling of basic volume plasma polarization exciton and basic hyperbolic phonon polarization exciton. This mode is then modulated by high refractive index nanowires [9]. Zulqarnain, M et al. designed an air and glass-modified graphene rectangular waveguide for surface wave propagation. The research employs boundary conditions to analyze the characteristics of surface waves and examines the impacts of chemical potential (CP) on dispersion curves. The results are logically presented through graphical representations of the improved graphene rectangular waveguide [39]. Additionally, Liu et al. presented a polarization-insensitive Gr waveguide with bilayer graphene [40].

Chemical vapor deposition (CVD) can be utilized to produce 2D films on various substrates in experimental settings. This technique has been successfully employed to produce large-area 2D films with adjustable thicknesses [41]. Furthermore, both graphene, SiO_2_ and hBN can be mechanically transferred onto different material substrates. Laser patterning has been extensively employed for several decades to fabricate 2D and 3D structures in polymers, metal surfaces, and bulk materials [42].

In this paper, we propose a planar hyperbolic phonon polaritons waveguide (HPhPs-WG) including hBN/SiO_2_/Gr/hBN and gold substrate to enhance optical performance with minimal propagation loss in the mid-IR spectrum. The investigation focuses on the impact of the hBN rib, geometry parameters of the HPhPs-WG, and CP of Gr on the guiding properties of the structures. The proposed structure can be utilized as a tunable low-loss and high-performance HPhPs-WG in the mid infrared range spectral range.

## 2 Geometry and formalism

**Fig. 1:**
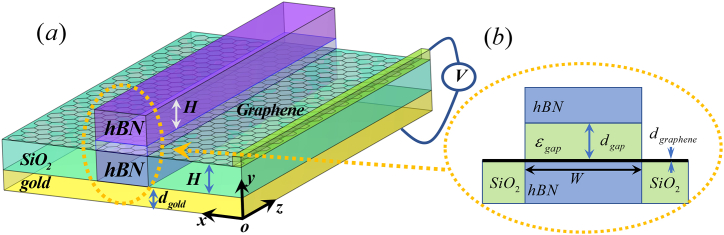
Schematics of the HPhPs-WG, (a)3D diagram and (b)cross-sectional view.

The three-dimensional schematic of hBN waveguide is depicted in Fig. 1. The geometry parameters of the HPhPs-WG are detailed in Table 1.

**Table 1:** Geometry parameters of the HPhPs-WG.

Name	Parametric description	Values
W	Width of the HPhPs-WG	150 nm
H	Thickness of the upper and bottom hBN layer	150 nm
d_gap_	Thickness of the gap of hBN rib	50 nm
d_gold_	Thickness of the gold layer	50 nm
d_graphene_	Thickness of the graphene layer	1 nm

The permittivity of air cladding and SiO_2_ part in the waveguide are $\varepsilon_{air}=1.0$ and $\varepsilon_{SiO_{2}}=2.3$. hBN is a natural hyperbolic metamaterial whose permittivity is a tensor. The transverse(*t*) and longitudinal(*l*) permittivity of hBN can be expressed as [43]

(1) $$\varepsilon_{i}\left(\omega \right)=\varepsilon_{\infty ,i}\left[1+\frac{\omega_{LO,i}^{2}-\omega_{TO,i}^{2}}{\omega_{TO,i}^{2}-\omega^{2}-i\omega \tau_{h,i}}\right]$$

where *i* = *t* or *l*. $\omega_{TO,i}$ and $\omega_{LO,i}$ are the frequencies of the pure transverse and pure longitudinal long optical phonons in *i*-direction, respectively. The angular frequency is *ω*. We have $\omega_{TO,t}=780cm^{-1}$, $\omega_{LO,t}=830cm^{-1}$ in the type I hyperbolic band (HB I) and $\omega_{TO,l}=1370cm^{-1}$, $\omega_{LO,l}=1610cm^{-1}$ in the type II hyperbolic band (HB II). $\varepsilon_{\infty ,i}$ is the high-frequency dielectric permittivity, $\varepsilon_{\infty ,l}=2.95$, $\varepsilon_{\infty ,t}=4.87$. $\tau_{h,i}$ is the damping constant, $\tau_{h,l}=4cm^{-1}$, $\tau_{h,t}=5cm^{-1}$ [43]. The permittivity of hBN is $\varepsilon =diag\left(\varepsilon_{x},\varepsilon_{y},\varepsilon_{z}\right)$, which correspond to the permittivity of $\varepsilon_{x}=\varepsilon_{l}$ and $\varepsilon_{y}=\varepsilon_{z}=\varepsilon_{t}$, respectively.

The permittivity of gold is $\varepsilon_{Au}=\varepsilon_{\infty ,Au}-\omega_{p}^{2}/\omega \left(\omega +i\tau_{Au}\right)$ [44], where $\varepsilon_{\infty ,Au}$ is the permittivity at the infinite frequency, $\omega_{p}$ is the bulk plasma. $\tau_{\text{Au}}$ is electron collision frequency. We set $\varepsilon_{\infty ,Au}=9.75$, $\omega_{p}=1.36\times 10^{6}rad/s$, and $\tau_{Au}=1.45\times 10^{14}rad/s$ [45]. The conductivity of Gr is [46]

(2) $$\sigma \left(\omega ,\Gamma ,\mu_{c},T\right)=\sigma_{\text{intra}}\left(\omega ,\Gamma ,\mu_{c},T\right)+\sigma_{\text{inter}}\left(\omega ,\Gamma ,\mu_{c},T\right)=\frac{ie^{2}k_{B}T}{\pi h^{2}\left(\omega -2i\tau_{G}^{-1}\right)}\left[\frac{\mu_{c}}{k_{B}T}+2\;\ln\left(e^{-\mu_{c}/k_{B}T}+1\right)\right]-\frac{ie^{2}}{4\pi h}\ln\left[\frac{2\left|\mu_{c}\right|-\left(\omega -2i\tau_{G}^{-1}\right)h}{2\left|\mu_{c}\right|+\left(\omega -2i\tau_{G}^{-1}\right)h}\right]$$

where $\tau_{G}$ is relaxation time, the reduced Planck's constant is ℏ, the electronic charge is e. The intraband conductivity is $\sigma_{\text{intra}}\left(\omega ,\Gamma ,\mu_{c},T\right)$ , the interband conductivity is $\sigma_{\text{inter}}\left(\omega ,\Gamma ,\mu_{c},T\right)$, the temperature in Kelvin is T = 300 k and $\tau_{G}^{-1}=2\times 10^{12}S^{-1}$. $\mu_{c}$ is the chemical potential and $k_{B}$ is the Boltzmann constant. The *E*_*F*_ can be adjusted extensively (from -1eV to 1eV) by applying a transverse electric field through a DC-biased gate. Gr's Fermi energy is

(3) $$E_{F}=\hbar v_{f}\sqrt{\frac{\pi \varepsilon_{r}\varepsilon_{0}V_{DC}}{eH}}$$

where $\varepsilon_{r}$ is the static permittivity of dielectric layer. $\varepsilon_{0}$ is the vacuum permittivity. The Fermi velocity of Gr varies inversely with its dielectric constant when the surrounding medium of Gr is altered. We set $v_{f}\approx 1.49\times 10^{6}m/s$ with bottom hBN layer [46]. The voltage is deemed to be feasible in both theory [47] and experiment [48]. The applied gate voltage is $V_{DC}$ [49]. According to Eq. (3), the gate voltages and the Fermi energies of Gr are shown in Table 2.

**Table 2:** Fermi energy and applied gate voltage.

*E*_*F*_ (*eV*)	V_DC_(V)	*E*_*F*_ (*eV*)	V_DC_(V)
0	0	0.6	50.89
0.1	8.45	0.7	59.39
0.2	16.94	0.8	67.89
0.3	25.45	0.9	76.38
0.4	33.94	1.0	84.88
0.5	42.44		

The equivalent relative permittivity of graphene is [50].

(4) $$\varepsilon_{g}=1+i\sigma_{g}/\left(\varepsilon_{0}\omega d_{gr}\right)$$

The propagation distance serves as an indicator of transmission loss in the waveguide. It is defined as [51].

(5) $$l_{p}=\frac{\lambda }{4\pi \left|\text{Im}\left(n_{eff}\right)\right|}$$

where Im (*n*_*eff*_) is imaginary part of the refractive index. The mode area is the ratio of the total electromagnetic energy to the peak energy density. The diffraction limited mode field area is $A_{0}=\lambda^{2}/4$ in the vacuum. The effective mode field area (*A*_*eff*_) is [52]

(6) $$A_{eff}=\frac{W_{m}}{W{\left(x,y\right)}_{\max}}=\frac{\iint W\left(x,y\right)dxdy}{\max\left(W\left(x,y\right)\right)}=\frac{\iint \frac{d\varepsilon \left(x,y\right)\omega }{d\omega }{\left|E\left(x,y\right)\right|}^{2}+\mu_{0}{\left|H\left(x,y\right)\right|}^{2}dxdy}{\max\left(\frac{d\varepsilon \left(x,y\right)\omega }{d\omega }{\left|E\left(x,y\right)\right|}^{2}+\mu_{0}{\left|H\left(x,y\right)\right|}^{2}\right)}$$

where *W*_*m*_ is the total electromagnetic energy density distribution in the waveguide, *W* (*x*,*y*) is the electromagnetic energy density. The peak energy of *W* (*x*,*y*) is *W* (*x*,*y*)_max_. Therefore, the figure of merit (*FOM*) is the quality factor. The *FOM* formula is [53].

(7) $$FOM=\frac{l_{p}}{2\sqrt{A_{eff}/\pi }}$$

The phase changes as a function of the refractive index real part (Re (*n*_*eff*_)) variation can be evaluated and expressed as

(8) $$\Delta \varphi =2\pi \text{Re}\left(n_{eff}\right)l/\lambda$$

where the operating wavelength is λ, *l* is the device length [50]. To analyze an optical phase shifter with the device length, we get $l=2\lambda /\text{Re}\left(n_{eff}\right)$ with Δφ=π.

When the propagation distance (*l*_*p*_) is larger than the device length (*l*), the optical phase shifter can be fabricated. The propagation loss is [54].

(9) $$\alpha =\frac{10\text{Im}\left(n_{eff}\right)4\pi }{\lambda_{0}\;\ln\;10}$$

where the imaginary parts of Im (*n*_eff_).

The optical response can be studied using the finite-difference time-domain (FDTD) simulations. The mode characteristics of the HPhPs-WG are simulated using COMSOL.

## 3 Mode characteristics and parameter optimizations

**Fig. 2:**
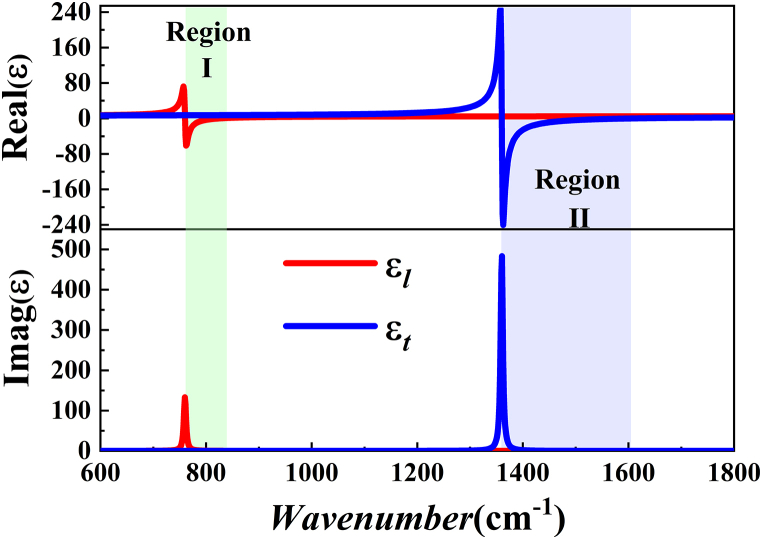
Real and imaginary parts of the permittivity components $\varepsilon_{t}$ and $\varepsilon_{l}$ of hBN varying with frequency.

The permittivity of hBN was illustrated in Fig. 2. A hBN crystal exhibits two hyperbolic frequency bands. Either of HB I in the range of 780.1 cm^−1^<ω < 829.9 cm^−1^ ($real\left(\varepsilon_{l}\right)<0$ and $\left(\varepsilon_{t}\right)>0$) and HB II in the range of 1360.0 cm^−1^< ω < 1609.8 cm^−1^ ($real\left(\varepsilon_{l}\right)<0$ and $real\left(\varepsilon_{t}\right)>0$) is illustrated color range.

**Fig. 3:**
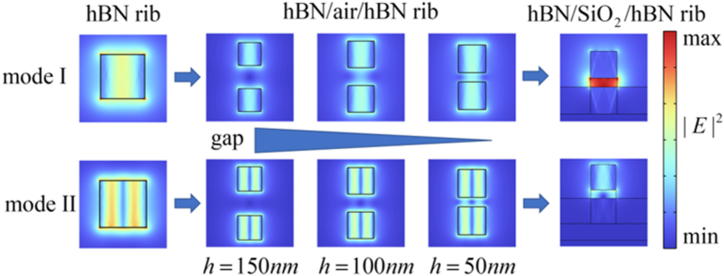
Electric field coupling steps of the proposed HPhPs-WG.

The HPhPs-WG mode is depicted in Fig. 3 and is elaborated upon as follows: (1) two HPhP eigenmodes are excited in single hBN rib. (2) HPhPs are excited on two hBN rib with a gap, with the electromagnetic fields of each hBN rib resembling that of single hBN rib when the gap larger than 100 nm. As the gap decreases, the coupling electromagnetic field is significantly enhanced in the gap. (3) For HPhPs-WG fabrication, a gold substrate, SiO_2_ part, and graphene layer with *E*_*f*_ = 0.1eV were added. The electric field energy is primarily located within the gap of the HPhPs-WG, arising from the coupling of HPhPs and graphene surface plasmons (GSPs). In mode I (MI), the electric field is centered in the middle of the gap and diminishes at the edges. In mode II (MII), the electric field is localized at the edges of the gap.

**Fig. 4:**
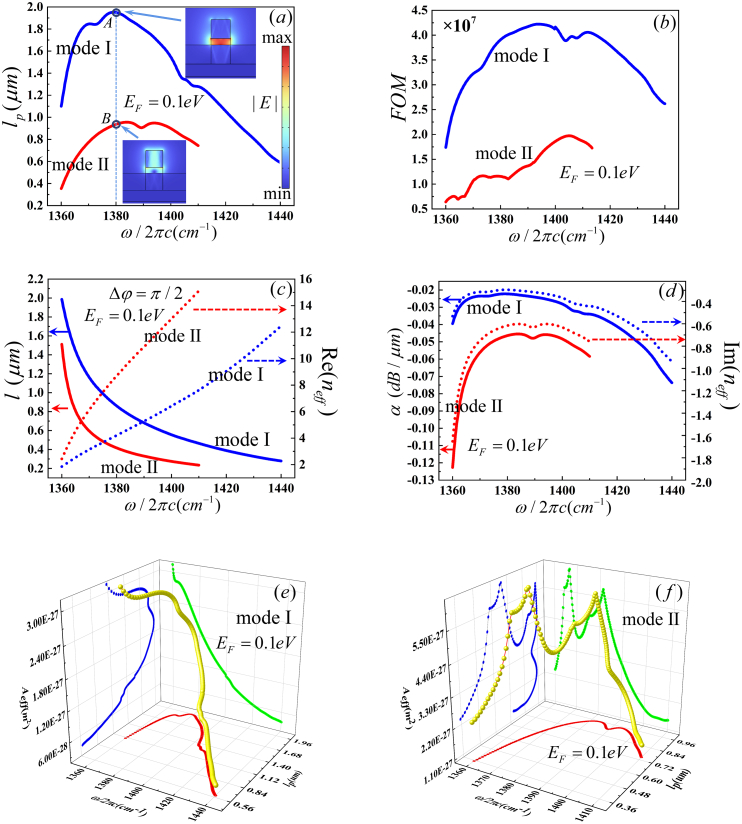
The guiding properties depend on the wavenumber of MI (blue line) and MII (red line). (a) propagation length (*l*_*p*_) with the inset shows the electric field distributions,(b) figure of merit (*FOM*), (c) device length (*l*) (solid line) with Re (*n*_*eff*_) (dot line), (d)propagation loss (*α*) (solid line)with Im (*n*_*eff*_) (dot line), (e) *l*_*p*_ and A_eff_ of MI, (f) *l*_*p*_ and A_eff_ of MII. (For interpretation of the references to color in this figure legend, the reader is referred to the Web version of this article.)

One of the key challenges of HPhPs-WG is to achieve a larger operation frequency bandwidth. Therefore, we numerically simulated several waveguide parameters, include the effective length (*l*_*p*_), figure of merit (FOM), device length (*l*) and propagation loss (*α*), with *H* = 150 nm, *W* = 150 nm and *E*_*F*_ = 0.1eV, as illustrated in Fig. 4. The HPhPs-WG MI and MII can be excited in frequency range of 1360∼1440 cm^−1^ and 1360∼1410 cm^−1^, respectively. Those frequency ranges are belonged to HB II with $\varepsilon_{x}>0$ and $\varepsilon_{y,z}<0$. According to Eq. (3) and Eq. (5), *l*_*p*_ and *FOM* of MI exceed those of MII. According to Fig. 4 (*a*) and (b), the maximum *l*_*p*_ of MI is approximately 1.95 μm at 1380 cm^−1^ with *FOM* is nearly 4.3 × 10^7^. In addition, the maximum *l*_*p*_ of MII is approximately 0.95 μm at 1383 cm^−1^ with *FOM* is nearly 1.2 × 10^7^. Furthermore, Fig. 4(c), reveals that the Re (*n*_*eff*_) increases monotonically and the device length decreases monotonically with increasing the frequency for the same mode. According to Eq. (8), the device length varies inversely with the Re (*n*_*eff*_). The device length for MI exceeds that of MII. For MI, *l*_*p*_ is larger than *l* from 1363 cm^−1^ to 1340 cm^−1^, which provides the working frequency range of the optical phase shifter. Base Eq. (9), the propagation loss is proportional to the Im (*n*_*eff*_). Im (*n*_*eff*_) increase in the lower frequency and then decrease in the higher frequency as shown in Fig. 4(d). Our calculations demonstrate that the HPhPs-WG exhibits the lowest propagation loss at 1380 cm^−1^ for MI and 1384 cm^−1^ for MII, as depicted in Fig. 4(d). The relationship between A_eff_, *l*_p_ and *f* of MI and MII are depicted in Fig. 4(e and f). For MI, A_eff_ increases as *l*_p_ increase. However, in 1360∼1380 cm^−1^, A_eff_ is larger than that of 1380∼1440 cm^−1^, as shown in the projection on the *l*_p_-A_eff_ plane in Fig. 4(e). Similarly, there is a consistent trend of A_eff_ increasing with an increase in *l*_p_. The relationship between A_eff_, *l*_p_ and the MII frequency is more intricate than MI. The maximin A_eff_ is approximately 6.30Х10^−27^ m^2^ at *l*_p_ = 0.67 μm at 1366.7 cm^−1^.

Table 3 shows important parameters such as *n*_eff_, *W*_m_, *W* (x,y)_max_ and *A*_*eff*_, which vary with the calculated effective refractive indexes of the eigenmodes. Base on Eq. (5), the propagation distance is influenced by Im (*n*_*eff*_). The |Im (*n*_*eff*_)| of MI is lower than that of MII, leading to a larger *l*_p_ for MI compared to MII at the same frequency. |*E*| of MI and MII are illustrated in the insets of Fig. 3(a). At points A and B, MI exhibits a stronger electric field than MII, concentrated in the center of the gap. Consequently, the peak energy of electromagnetic energy density (*W* (x,y)_max_) of MI surpasses that of MII.

**Table 3:** $n_{eff}$, $W{\left(x,y\right)}_{\max}$*,*$W{\left(x,y\right)}_{\max}$ and $A_{eff}$ of Fig. 4.

Point	mode	$n_{eff}$	$\begin{array}{l} W{\left(x,y\right)}_{\max}\\ \left(10^{-7}J/m^{2}\right) \end{array}$	$W_{m}\left(10^{-20}J\right)$	$A_{eff}\left(10^{-13}m^{2}\right)$
A	I	4.2610–0.32696i	4.8730	8.8664	1.8195
B	II	8.7176–0.64573i	0.96406	1.4270	1.4802

According electric field distributions, the total electromagnetic energy density of MI exceeds that of MII. According Eq. (6), *A*_*eff*_ of MI exceeds that of MII. Base on *W* (x,y)_max_ and *A*_*eff*_, the *FOM* of MI exceeds that of MII, as shown in Table 3. Furthermore, the Re (*n*_*eff*_) of MI is lower than that of MII, leading to a greater device length for MI compared to MII at 1380 cm^−1^. Moreover, the propagation loss of MI exceeds MII, as Im (*n*_*eff*_) of MI is less than that of MII. Ultimately, MI proves more favorable as a transport mode in the waveguide owing to its superior parameters.

**Fig. 5:**
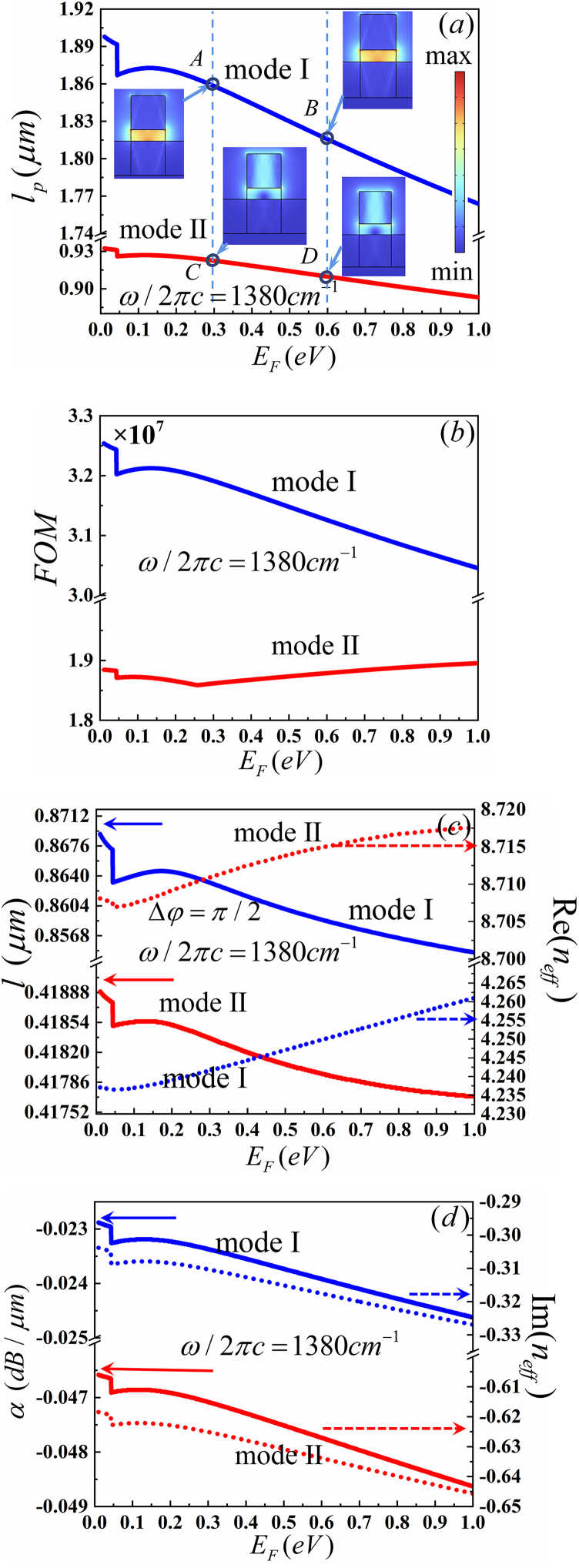
Dependence of the guiding properties on *E*_F_ of MI (blue line) and MII (red line) (a) propagation length (*l*_p_) with the inset shows the electric field distributions,(b) figure of merit (*FOM*), (c) device length (*l*) (solid line) with Re (*n*_*eff*_) (dot line) and (d)propagation loss (α) (solid line)with Im (*n*_*eff*_) (dot line)

Other challenges of HPhPs-WG are the waveguide parameters can be modulated by changing the physical parameters, such as gate voltage. Previous researches have shown that the HPhPs are sensitive to the local environment [55]. So, we have set graphene on hBN and SiO_2_ substrate, as shown in Fig. 1. Those waveguide parameters can be modulated by changing the *E*_F_. Fig. 5(*a*, *b*) illustrates that the *l*_*p*_ and *FOM* of MI exceeds that of MII at 1380 cm^−1^. The increase of the *E*_*F*_ from 0.1 to 1.0eV results in a decrease of the *l*_*p*_ from 1.90 to 1.76 and *FOM* from 3.25 × 10^7^ to 3.04 × 10^7^ of MI. The maximum *l*_*p*_ of MI is approximately 0.93 μm at 0.1eV and that of MII is nearly 0.89 μm at 0.1eV. When the gate voltage is low, the *E*_F_ is below the threshold (*E*_*F*_ <ℏω/2 = 0.48eV), causing the absorption of external photons and resulting in interband transitions [56]. This is depicted by the rapid changes in the curves shown in Fig. 5.

According to Eq. (8), the device length is inversely proportional to the Re (*n*_*eff*_). The Re (*n*_*eff*_) increases monotonically and the device length decreases monotonically with increasing the frequency for the same mode, as shown in Fig. 5(c). Furthermore, *l*_*p*_ is larger than *l* for both MI and MII. This implies that the optical phase shifter can be utilized within the gate voltage range from 1.84 V to 184.4 V base on Eq. (3). According to Eq. (9), the propagation loss is proportional to the Im (*n*_*eff*_). Im (*n*_*eff*_) decreases monotonically with increasing frequency, as shown in Fig. 5(d). The HPhPs-WG exhibits the lowest propagation loss at 0.5eV for MI and 0.3eV for MII, as depicted in Fig. 5(d).

Some important parameters, such as *n*_*eff*_, *W*_m_, *W* (x,y)_max_ and *A*_*eff*_, are showed in Table 4. It is observed that the propagation distance varies with Fermi energy. For MI, the |Im (*n*_*eff*_)| of 0.3eV exceeds that of 0.6eV, thus *l*_p_ of point A is larger than point B in the same frequency. The same characteristics are observed in point C and point D for MII. The electric field distributions of MI and MII at 0.3eV and 0.6eV are shown in the insets of Fig. 3(a), respectively. It is clearly seen that the electric field intensity of MI exceeds that of MII, as shown at point A and C of 0.3eV or point B and D of 0.6eV. However, there is no significant difference in the electric field distributions of 0.3eV and 0.6eV in the same mode. Base on Table 4, *W*_*m*_ and *W* (x,y)_max_ of 0.3eV is larger than that of 0.6eV. Additionally, *A*_*eff*_ of MI is grearer than MII. Hence, the *FOM* for MI exceeds that of MII, as shown in Fig. 5(b). It has been observed that Re (*n*_*eff*_) for MI is smaller than MII at same *E*_F_. Consequently, the device length of MI exceeds that of MII. Additionally, propagation loss for MI surpasses MII, given that Im (*n*_*eff*_) for MI is smaller than II.

**Table 4:** $n_{eff}$, $W{\left(x,y\right)}_{\max}$*,*$W{\left(x,y\right)}_{\max}$ and $A_{eff}$ of Fig. 5.

Point	mode	$n_{eff}$	$\begin{array}{l} W{\left(x,y\right)}_{\max}\\ \left(10^{-7}J/m^{2}\right) \end{array}$	$W_{m}\left(10^{-20}J\right)$	$A_{eff}\left(10^{-13}m^{2}\right)$
A	I	4.2416–0.31032i	4.8301	8.8361	1.8294
B	I	4.2499–0.31761i	4.8502	8.8501	1.8247
C	II	8.7109–0.62512i	0.90850	1.4142	1.5567
D	II	8.7150–0.63387i	0.93434	1.4213	1.5212

**Fig. 6:**
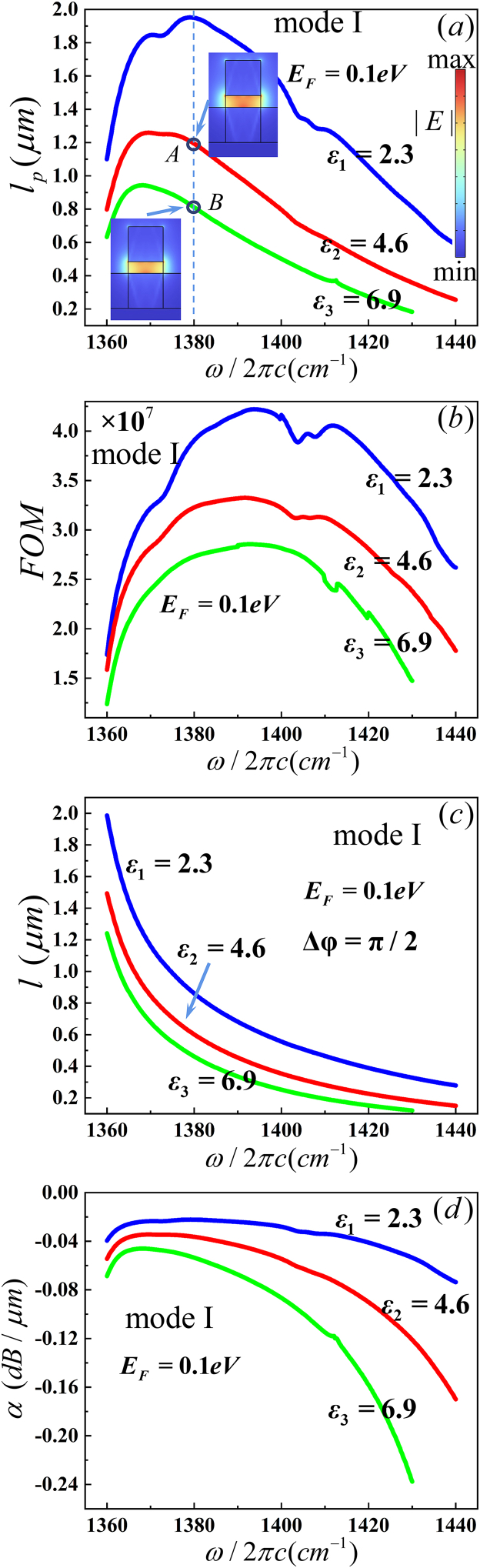
Dependence of the guiding properties on the gap (a) propagation length (*l*_*p*_), the inset shows the electric field distributions, (b) figure of merit (*FOM*), (c) device length (*l*) and (d)propagation loss (*α*).

The dependences of the waveguide parameters on several key structure parameters were investigated. The electric fields were designed to focus in the gap of upper and under hBN ribs, and the permittivity of the filling medium in the gap was found to have a significant influence on the waveguide parameters while keeping other structure parameters constant. Three types of permittivity were set in the following simulation, where $\varepsilon_{1}=\varepsilon_{SiO_{2}}=2.3<\varepsilon_{⊥,//}$, $\varepsilon_{\infty ,//}<\varepsilon_{2}=4.6<\varepsilon_{\infty ,⊥}$ and $\varepsilon_{3}=6.9>\varepsilon_{\infty ,⊥}$. The permittivity $\varepsilon_{2}$ and $\varepsilon_{3}$ was given by Si_3_N_4_/SiC composite material with different volume ratios. Base on the above analyses, only MI was simulated in following discussions. According to Table 3, Table 4, it is clearly seen that the |Im (*n*_*eff*_)| increases with increasing permittivity. As the curves in Fig. 6(a) and (b), *l*_*p*_ and *FOM* decreases with increasing permittivity of the gap in all frequency range. *l*_*p*_ has a maximum value near 1380 cm^−1^ with SiO_2_ in the gap and near 1370 cm^−1^ with $\varepsilon_{2}$ or $\varepsilon_{3}$ in the gap, as displayed in Fig. 6(a). The electric field distributions of MI and MII are illustrated in the insets of Fig. 6(a). According to Figs. 4(a)–Fig. 6(a), Table 3, Table 5, it is clear that the electric field intensity and *W* (x,y)_max_ increases with increasing permittivity. *W*_m_ decreases with increasing permittivity as shown in Table 5. *FOM* has a maximum value near 1390 cm^−1^ for fixed permittivity for $\varepsilon_{1}$, $\varepsilon_{2}$ and $\varepsilon_{3}$ , as illustrated in Fig. 6(b). From Fig. 6(c), it is noted that the device length decreases steadily as the frequency increases. *l*_*p*_ is larger than *l* with frequency larger than 1363.6 cm^−1^ for $\varepsilon_{1}$, 1364 cm^−1^ for $\varepsilon_{2}$ and 1364.5 cm^−1^ for $\varepsilon_{3}$. The optical phase shifter will be used in these frequency ranges. We find that Re (*n*_*eff*_) increases with increasing permittivity, as shown in Table 5. Thus, the device length decreases with increasing permittivity. The propagation loss increases with the increase of the frequency, and it shows the lowest propagation loss at 1380 cm^−1^ for $\varepsilon_{1}$ and 1368 cm^−1^ for $\varepsilon_{2}$ and $\varepsilon_{3}$, as shown in Fig. 6(d). The propagation loss decreases with the increase of the permittivity, because the |Im (*n*_*eff*_)| increases with increasing permittivity. By comparing the characteristics of waveguide parameters, SiO_2_ was set in the gap of our simulation.

**Table 5:** $n_{eff}$, $W{\left(x,y\right)}_{\max}$*,*$W{\left(x,y\right)}_{\max}$ and $A_{eff}$ of Fig. 6.

Point	mode	$n_{eff}$	$\begin{array}{l} W{\left(x,y\right)}_{\max}\\ \left(10^{-7}J/m^{2}\right) \end{array}$	$W_{m}\left(10^{-20}J\right)$	$A_{eff}\left(10^{-13}m^{2}\right)$
A	I	6.0446–0.47890i	15.242	16.9963	1.1151
B	I	7.8749–0.70561i	17.381	15.4082	0.88650

**Fig. 7:**
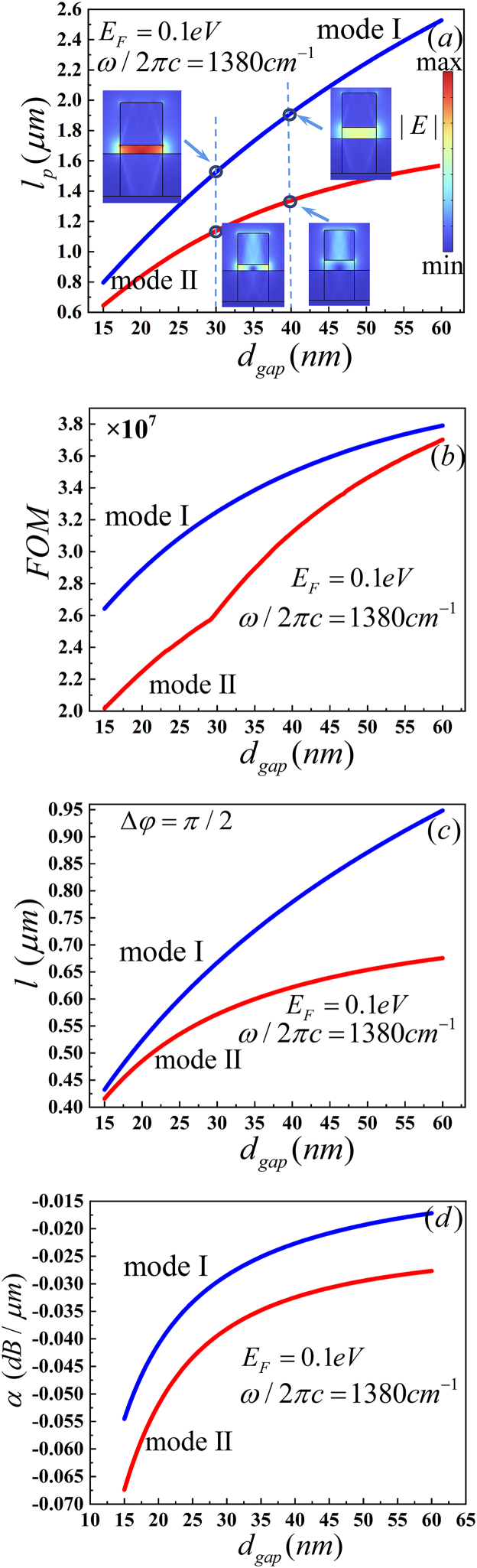
Dependence of the guiding properties on *d*_gap_ (nm) (a) propagation length (*l*_*p*_), the inset shows the electric field distributions, (b) figure of merit (*FOM*), (c) device length (*l*) and (d)propagation loss (*α*).

The thickness of the gap is an importance factor. We varied the thickness from 15 to 60 nm to investigate MI and MII. *l*_p_ and *FOM* increase with thickness increases, as shown in Fig. 7(a)and (b). Furthermore, *l*_p_ and *FOM* of MI are notably greater than those of MII. *l* is monotonically decreasing and *α* is monotonically increasing with the increasing thickness for SiO_2_ in the gap at 1380 cm^−1^, as illustrated in Fig. 7(c) and (d) *l*_*p*_ is greater than *l* for both MI and MII in all thickness. It means that we can design an optical phase shifter with a gap smaller than 60 nm. Furthermore, *α* of MI is less than that of MII. *l*_*p*_ and *α* have a small change with the thickness larger than 50 nm. According to Table 6, |Im (*n*_*eff*_)| and Re (*n*_*eff*_)of MI are less than those of MII, and |Im (*n*_*eff*_)| and Re (*n*_*eff*_) decrease with increasing thickness in the same mode. So, the *l*_p_ and *l* increase and *α* decreases with increasing gap. The electric field distributions of MI and MII are illustrated in the insets of Fig. 7(a). Important parameters are showed in Table 6. The peak energy of electromagnetic energy density *W* (x,y) _*max*_ and *W*_m_ of MI is larger than MII. According electric field distributions, *A*_*eff*_ of MI is than MII. Base on *W* (x,y)_max_, *W*_m_ and *A*_*eff*_, the *FOM* of MI is greated than MII. Therefore, we set 50 nm of the thickness in our simulation.

**Table 6:** *n*_*eff*_, max (W (x,y)) and *A*_*eff*_ of Fig. 7.

Point	mode	$n_{eff}$	$\begin{array}{l} W{\left(x,y\right)}_{\max}\\ \left(10^{-7}J/m^{2}\right) \end{array}$	$W_{m}\left(10^{-20}J\right)$	$A_{eff}\left(10^{-13}m^{2}\right)$
A	I	5.4191–0.43393i	5.2483	6.7871	1.2932
B	I	4.6830–0.34828i	4.7974	7.7252	1.6103
C	II	9.1614–0.70204i	1.3006	1.2729	0.97872
D	II	8.8590–0.64881i	1.0447	1.2754	1.2209

**Fig. 8:**
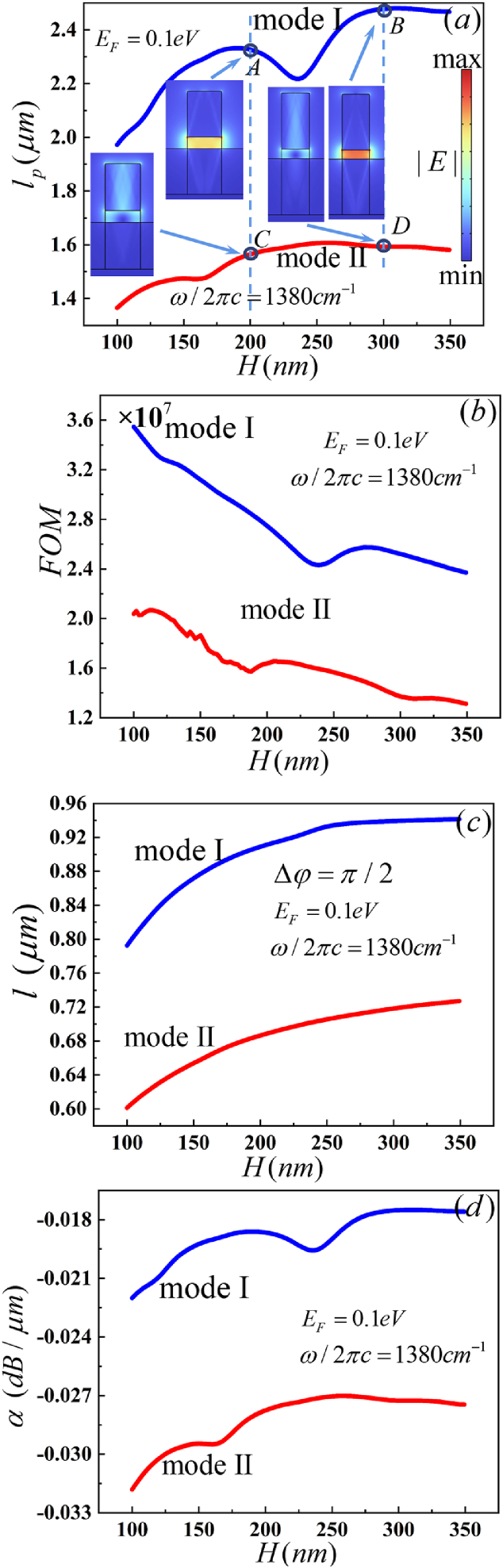
Dependence of the guiding properties on H (nm) (a) propagation length (*l*_*p*_), the inset shows the electric field distributions, (b) figure of merit (*FOM*), (c) device length (*l*) and (d)propagation loss (*α*).

The thickness and width of the hBN rib are also importance factors of the HPhPs-WG. *l*_*p*_ increases and *FOM* decreases with the hBN rib thickness increases for *W* = 150 nm at 1380 cm^−1^, as shown in Fig. 8(a and b). MI has a larger *l*_*p*_ and *FOM* than MII. When H > 300 nm, the *l*_*p*_ of the MI and MII remains unchanged, but *FOM* is decreasing with increasing thickness. *l* is monotonically increasing with the thickness increasing, as illustrated in Fig. 8(c) *l*_*p*_ exceeds that of *l* of both MI and MII. *α* is decreasing with increasing thickness, as shown in Fig. 8(d) *α* of MI is lower than MII. Base on Table 7, the |Im (*n*_*eff*_)| of MI is lower than MII and increases with the hBN rib thickness increasing in the same mode. So, *l*_p_ increases and *α* decreases with the increasing thickness. Re (*n*_*eff*_) of MI is less than that of MII and decreases with the increasing thickness in the same mode. Thus, *l* increases with the increasing thickness. The electric field distributions of MI and MII are illustrated in the insets of Fig. 8(a). The *W* (x,y)_max_ and *W*_*m*_ (x,y) of MI is larger than MII. According to the electric field distributions, the *A*_*eff*_ of MI is smaller than MII. Base on *W* (x,y)_max_, *W*_*m*_ (x,y) and *A*_*eff*_, the *FOM* of MI is greated than MII. After comparing the characteristics of waveguide parameters, we set the thickness of 150 nm in our simulation.

**Table 7:** *n*_*eff*_, max (W (x,y)) and *A*_*eff*_ of Fig. 8.

Point	mode	$n_{eff}$	$\begin{array}{l} W{\left(x,y\right)}_{\max}\\ \left(10^{-7}J/m^{2}\right) \end{array}$	$W_{m}\left(10^{-20}J\right)$	$A_{eff}\left(10^{-13}m^{2}\right)$
A	I	4.0012–0.28423i	5.4146	11.7578	2.1715
B	I	3.8453–0.29507i	6.5986	16.1863	2.4530
C	II	8.4637–0.60333i	0.99129	1.5933	1.6074
D	II	8.2510–0.61148i	1.2005	1.9869	1.6551

**Fig. 9:**
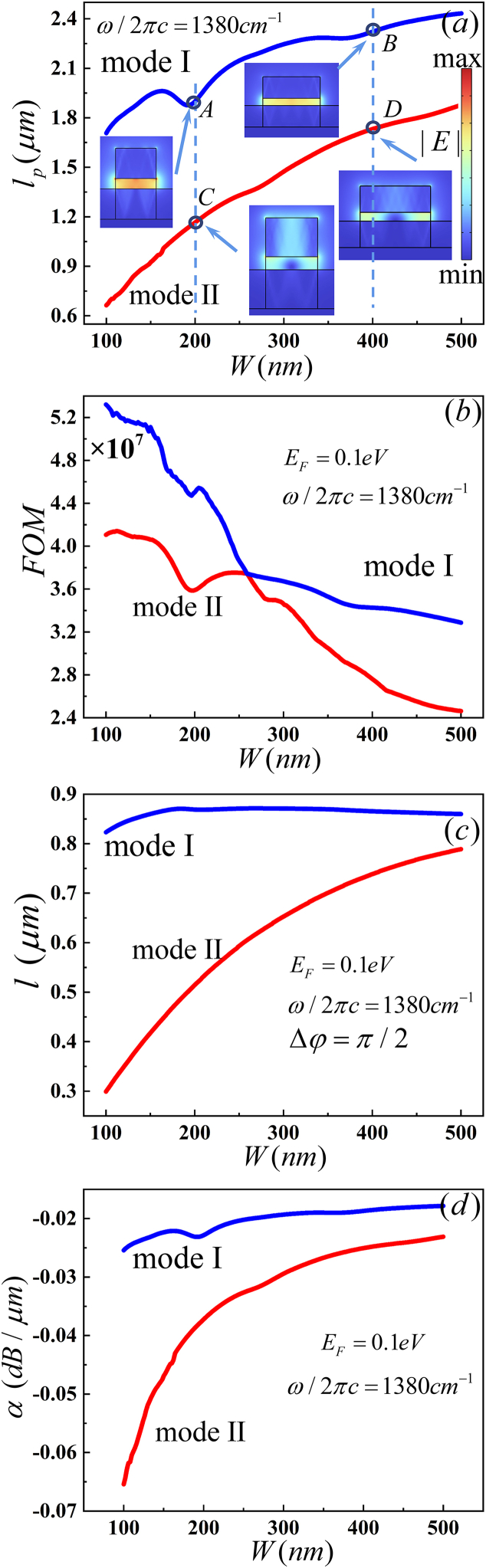
Dependence of the guiding properties on W (nm) (a) propagation length (*l*_p_), the inset shows the electric field distributions, (b) figure of merit (*FOM*), (c) device length (*l*) and (d)propagation loss (α).

In addition, the width of hBN ribs play key roles for the HPhPs-WG. *l*_*p*_ of MI is larger than MII with W increasing, as shown in Fig. 9(a). *FOM* decreases with the width increasing and MI and MII have the same *FOM* near *W* = 260 nm, as shown in Fig. 9(b) *l* of MI remains unchanged and *l* of MII is increases as W increasing, as illustrated in Fig. 9(c). As *l*_*p*_ exceeds that of *l*, the optical phase shifter can be designed in the width range of 100 nm–500 nm. *α* of MI is less than MII, as shown in Fig. 9(d). According to Table 8, the |Im (*n*_*eff*_)| of MI is less than MII at same width. The |Im (*n*_*eff*_)| decreases with the width increasing in the same mode, resulting in an increase in *l*_p_ and a decrease in *α*. The Re (*n*_*eff*_) of MI is less than II in the same width and decreases with the width increasing in the same mode, leading to an increase in *l*. The electric field distributions of MI and MII are shown in the insets of Fig. 9(a). The *W* (x,y)_max_ and *W*_*m*_ (x,y) of MI exceeds that of MII. According to the electric field distributions, the *A*_*eff*_ of MI is less than MII. Therefore, the *FOM* of MI exceeds that of MII. After comparing the characteristics of waveguide parameters, we set the width of 150 nm in our simulation.

**Table 8:** *n*_*eff*_, max (W (x,y)) and A_*eff*_ of Fig. 9.

Point	mode	$n_{eff}$	$\begin{array}{l} W{\left(x,y\right)}_{\max}\\ \left(10^{-7}J/m^{2}\right) \end{array}$	$\begin{array}{l} W_{m}\left(x,y\right)\\ \left(10^{-20}J\right) \end{array}$	$A_{eff}\left(10^{-13}m^{2}\right)$
A	I	4.1838–0.3143i	10.2190	20.5473	2.0107
B	I	4.2045–0.2534i	12.7603	28.002	2.1945
C	II	7.0431–0.5084i	2.8376	4.5872	1.6166
D	II	4.9064–0.3430i	5.4062	12.7591	2.3601

**Fig. 10:**
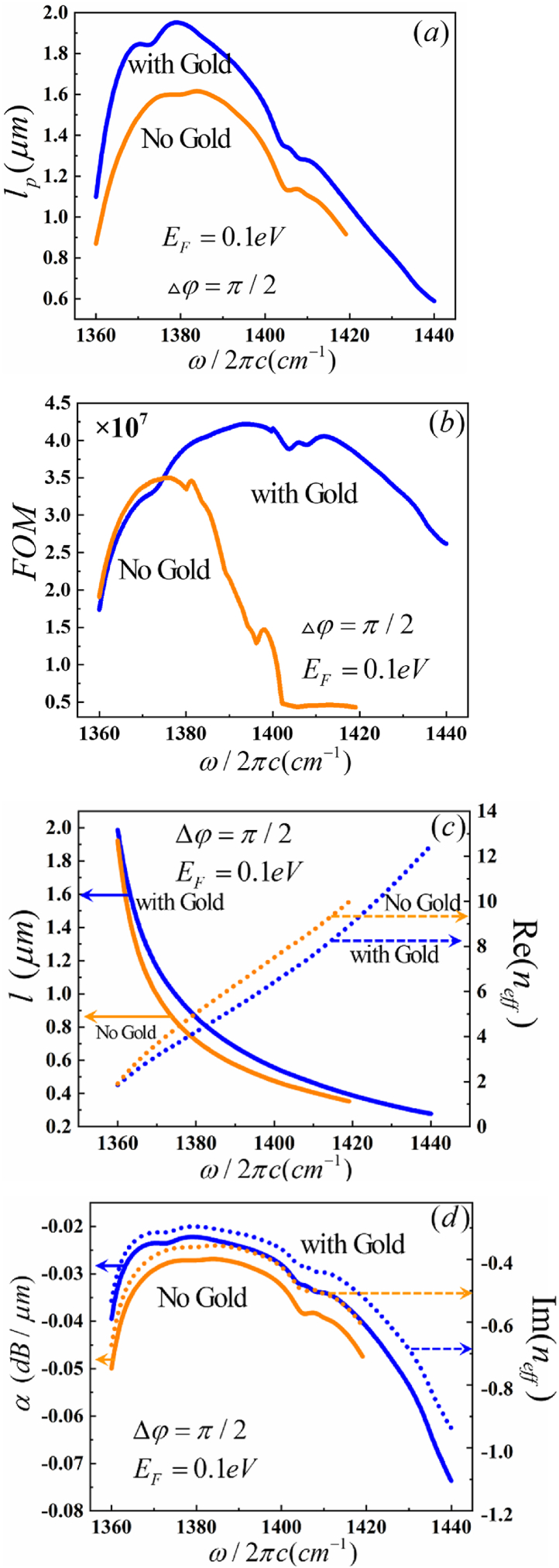
Dependence of the guiding properties on gold substrate of MI. (a) propagation length (*l*_p_), the inset shows the electric field distributions, (b) figure of merit (*FOM*), (c) device length (*l*) and (d)propagation loss (α)

To investigate the influence of the gold substrate within the HPhPs-WG, we simulate *l*_*p*_, FOM, *l* and *α*, as shown in Fig. 10. With increasing requency, *l*_*p*_ and FOM of HPhPs-WG were found to be larger than those of the structure without a gold substrate, as depicted in Fig. 10 (a, b). The frquency range of MI of HPhPs-WG was wider than the structure without a gold substrate. Moreover, with increasing frequency, *l* and *α* of the structure without a gold substrate were smaller than those of HPhPs-WG, as illustrated in Fig. 10 (c, d). The gold layer acts as a substrate to support the optical waveguide, and simultaneously blocks the downward propagation of electromagnetic waves.

The comparison of the constitutive materials, operating overlength, unit cell dimensions, *FOM*, propagation distance, and propagation loss of the HPhPs-WG with some others’ work is presented in Table 9, which demonstrates that the HPhPs-WG has a superior *FOM* and propagation loss.

**Table 9:** The comparison between our work and references.

Constitutive Materials	Operating Wavelength	Unit Cell Dimensions	*FOM*	*l*_*p*_	*α* (dB/μm)	Photo	Ref.
Si_3_N_4_/hBN/Graphene/SiO_2_	800–1600 nm	65nm/700nm/200 μm	2.715 × 10^13^	1.2965 μm	0.17		[11]
Si_3_N_4_/Graphene/Na/SiO_2_	2000 nm	350nm/150nm/200 nm	1 × 10^5^	1 × 10^5^nm	0.69		[57]
Graphene/Si/hBN/Au	1200–1700 nm	500nm/250 nm /150nm/50 nm	＞1 × 10^5^	\	0.01		[38]
Ag/Graphene/Al_2_O_3_/Ag	1310 nm	60nm/10 nm	1.63 × 10^11^	\	0.17		[56]
Si/Ag/SiO_2_/TMD/Ag	1550 nm	10nm/200nm/5 nm	<10^5^	10^3^μm	0.633		[53]
Si/hBN/Graphene/SiO_2_	7140 nm	300 nm	100–300	1–3 μm	0.01373		[10]
GaAs/MgF_2_/Graphene/hBN	7100 nm	50 nm	20–60	0.2–0.6 μm	0.021		[58]
SiO_2_/Au/Graphene/electrode	9993.1–11992 nm	100nm/30nm/20 nm	15	20 μm	1.12		[59]
Au/SiO_2_/SiO_2_	7400–8200 nm	100nm/100nm/100 nm	\	2.258 μm	2.2		[60]
AvdWM/spacer/G	-1-300 cm	200 nm	20	\	\		[61]
SiC/BP/BaF_2_	8-14 μm	100nm/150 nm	\	\	\		[62]
SiO_2_/SiC/MoO_3_	8-22 μm	1.5 μm/150nm/1.5 μm	\	\	\		[63]
hBN/SiO_2_/hBN/Au	6940–7350 nm	150nm/50nm/150 nm	4 × 10^7^	2 μm	0.0225		Our work

## 4 Conclusion

This paper presents a mid infrared range waveguide composed of hBN/SiO_2_/graphene/hBN ribs. By coupling the HPhP of the upper of the hBN rib with the HPhP of the lower hBN rib and GSPs in the middle dielectric rib, we found that the hyperbolic phonon polaritons waveguide (HPhPs-WG) model follows the dependences of the effective length (*l*_*p*_), figure of merit (*FOM*), device length (*l*) and propagation loss (*α*) on the applied gate voltage of graphene layer. The phenomenon is caused by the alterable equivalent relative permittivity dielectric of graphene layer in the second reststrahlen band (1360.02 cm^−1^<ω < 1609.98 cm^−1^) of hBN. Through optimizing geometry parameters and dielectric permittivity, the simulation results demonstrate a high *FOM* of 4.0 × 10^7^. We believe that the HPhPs-WG can be utilized as a platform for a variety of the mid infrared range photonic and optoelectronic devices.

## Data availability statement

Data will be made available on request.

## Additional information

No additional information is available for this paper.

## Funding statement

High-level Scientific Research Foundation for the introduction of talent of Guangzhou Maritime University, 10.13039/501100013772Harbin Normal University Master's Innovation Project (HSDSSCX2022-49, HSDSSCX2022-53, HSDSSCX2023-16); 10.13039/501100015341Key Laboratory of Engineering Dielectrics and Its Application (Harbin University of Science and Technology), 10.13039/100018901Ministry of Education (KFM202005, KF20171110); Postgraduate course construction project; 10.13039/501100005046Natural Science Foundation of Heilongjiang Province (LH2020A014); 10.13039/100014717Natural Science Foundation of China (11104050, 11204056).

## CRediT authorship contribution statement

**Zhou Sheng:** Writing – review & editing, Writing – original draft, Data curation, Conceptualization. **Liu Yue:** Writing – original draft, Software. **Yue Zhao:** Software, Data curation. **Gao Jin:** Data curation. **Qiang Zhang:** Methodology, Conceptualization. **Shufang Fu:** Writing – review & editing, Software. **Xiangguang Wang:** Data curation. **Xuan Wang:** Conceptualization. **Xuanzhang Wang:** Conceptualization.

## Declaration of generative AI and AI-assisted technologies in the writing process

During the preparation of this work the author(s) used Monica in order to improve readability and language. After using this tool/service, the author(s) reviewed and edited the content as needed and take(s) full responsibility for the content of the publication.

## Declaration of competing interest

The authors declare the following financial interests/personal relationships which may be considered as potential competing interests:

Sheng zhou reports financial support was provided by Guangzhou Maritime University.
